# Explainable AI for Material Property Prediction Based on Energy Cloud: A Shapley-Driven Approach

**DOI:** 10.3390/ma16237322

**Published:** 2023-11-24

**Authors:** Faiza Qayyum, Murad Ali Khan, Do-Hyeun Kim, Hyunseok Ko, Ga-Ae Ryu

**Affiliations:** 1 Department of Computer Engineering, Jeju National University, Jeju-si 63243, Republic of Korea; faizaqayyum@jejunu.ac.kr (F.Q.); muradali@stu.jejunu.ac.kr (M.A.K.); 2 Center of Materials Digitalization, Korea Institute of Ceramic Engineering and Technology, Jinju-si 52851, Republic of Korea; hko@kicet.re.kr

**Keywords:** material, ceramic, TabNet, machine learning, deep learning, explainable artificial intelligence, Shapely

## Abstract

The scientific community has raised increasing apprehensions over the transparency and interpretability of machine learning models employed in various domains, particularly in the field of materials science. The intrinsic intricacy of these models frequently results in their characterization as “black boxes”, which poses a difficulty in emphasizing the significance of producing lucid and readily understandable model outputs. In addition, the assessment of model performance requires careful deliberation of several essential factors. The objective of this study is to utilize a deep learning framework called TabNet to predict lead zirconate titanate (PZT) ceramics’ dielectric constant property by employing their components and processes. By recognizing the crucial importance of predicting PZT properties, this research seeks to enhance the comprehension of the results generated by the model and gain insights into the association between the model and predictor variables using various input parameters. To achieve this, we undertake a thorough analysis with Shapley additive explanations (SHAP). In order to enhance the reliability of the prediction model, a variety of cross-validation procedures are utilized. The study demonstrates that the TabNet model significantly outperforms traditional machine learning models in predicting ceramic characteristics of PZT components, achieving a mean squared error (MSE) of 0.047 and a mean absolute error (MAE) of 0.042. Key contributing factors, such as d33, tangent loss, and chemical formula, are identified using SHAP plots, highlighting their importance in predictive analysis. Interestingly, process time is less effective in predicting the dielectric constant. This research holds considerable potential for advancing materials discovery and predictive systems in PZT ceramics, offering deep insights into the roles of various parameters.

## 1. Introduction

Within the field of materials science, the precise forecasting of attributes of ceramic materials is regarded as a crucial undertaking with extensive ramifications [1]. The capacity to predict these properties is essential for the progression of materials exploration and design methodologies. The use of ceramics in several fields, including electronics, healthcare, and energy systems, is significantly influenced by advancements and breakthroughs in this domain [2]. There is an increasing demand for the integration of artificial intelligence (AI) applications in the simulation and exploration of innovative materials [3]. The application of AI analysis in materials design is expected to yield innovative materials while reducing the time and resources required for development. However, it is important to note that the scientific community has recognized many limitations linked to the implementation of sophisticated materials discovery and artificial intelligence (AI) approaches in this particular domain [2]. Computational simulation has several obstacles, and the studied materials require high-performance-index properties [4]. Therefore, it is imperative to conduct sophisticated research on materials that combine artificial intelligence techniques with experimental methods in order to develop a comprehensive understanding of the relationships between input parameters and performance indices [5,6,7]. The field of machine learning originated from the desire to achieve artificial intelligence [8]. During the 1950s, many symbolic methods were employed in order to tackle the difficulty of machine knowledge acquisition [9]. Subsequently, a comprehensive investigation was undertaken to explore methodologies based on the notion of connection, such as neural networks and perceptron [10]. Subsequently, a multitude of techniques rooted in statistical learning theory (SLT), including support vector machines (SVMs) [11] and decision trees (DTs) [12], were introduced. There is a significant amount of interest in academic and industry sectors regarding various advanced machine techniques, with a special focus on deep learning methods for analyzing large datasets. Machine learning functions as a mechanism for automating the creation of analytical models. Machine learning enables computers to discover hidden insights without explicit programming guidance by utilizing algorithms that iteratively learn from data [13]. It employs previous computations to produce dependable and replicable judgments and outcomes. As a result, it has significantly contributed to various fields, such as speech recognition, image recognition [14], bioinformatics [15], information security [16], and natural language processing (NLP) [17].

The utilization of machine learning in materials science first appeared during the 1990s. During this time, several methods, such as symbolic approaches and artificial neural networks (ANNs), were employed to predict corrosion behavior, as well as tensile and compressive strength in ceramic-matrix composites [18,19,20]. Subsequently, the field of machine learning has found extensive application in various domains of materials science, encompassing the investigation of novel materials, as well as the prognostication of material properties. The selection of an appropriate machine learning algorithm is a critical step in developing a machine learning system, as it substantially influences the accuracy of predictions and the system’s ability to generalize [21]. Probability estimate techniques are primarily utilized in the field of novel materials discovery [2]. In addition, the utilization of regression, clustering, and classification methods is prevalent in the prediction of material properties at both macroscopic and microscopic scales. In addition, machine learning techniques are commonly combined with other intelligent optimization algorithms [22,23], such as Genetic Algorithms (GAs), Simulated Annealing Algorithms (SAAs), or Particle Swarm Optimization (PSO) algorithms, particularly to optimize model parameters. Moreover, these optimization techniques can be employed to tackle complex optimization tasks, including the optimization of spatial configurations and material attributes. Isayev et al. (2019) [24] presented a computational tool called Property-Labelled Materials Fragments (PLMF). This tool was specifically developed to facilitate the construction of machine learning models for the prediction of the properties of inorganic crystals. The initial step in the PLMF method involves filtering characteristics that exhibit low variance and strong correlation, resulting in the creation of a feature vector. The classification of a potential material as either a metal or an insulator, along with the prediction of the band-gap energy in the case of an insulator, is accomplished through the utilization of the gradient boosting decision tree (GBDT) technique [25]. The computational method known as Property-Labelled Materials Fragments (PLMF), developed by Isayev et al. [24], was built specifically to create machine learning models to predict the properties of inorganic crystals. In the PLMF approach, the initial step involves filtering characteristics that exhibit low variance and strong correlation to generate a feature vector [25].

Lundberg and Lee (2019) [26] made a significant contribution to the field by employing the Shapley additive explanations (SHAP) method to forecast the properties of ceramic materials, with a particular focus on transparent frameworks. Xie and Grossman [27] conducted a thorough analysis of the shortcomings present in existing methodologies, which aligns with our objective of improving the accuracy of prediction models. Schmidt and Lipson [28] conducted a comprehensive examination of the utilization of machine learning techniques in the prediction of diverse ceramic material properties. The scholarly works by Ward et al. [29] provide a thorough overview, encompassing a range of topics such as general-purpose applications, precise thermal conductivity predictions, and the wider implications of deep learning in materials science. The study [30] utilized machine learning techniques on quantum computations to enhance the efficiency of material property predictions. The approach involves generating decision rules that rely on chemo-structural or electronic fingerprints. By utilizing these rules, the predictions are not only rapid but also highly precise. Consequently, this methodology expedites the process of discovering novel materials. Another study [31] showcased the application of machine learning techniques for predicting crucial characteristics of organic photovoltaic materials, including power conversion efficiency and molecular orbital energies. This process expedites the preliminary evaluation to develop efficient and economically viable solar cell designs in the context of green energy applications. The work [32] introduced MIPHA and rMIPHA, machine learning algorithms designed for the prediction and inverse analysis of steel characteristics and microstructures. These tools provide satisfactory performance and continuous enhancements, indicating possibilities for future microstructure-to-processing inverse analysis. The study [33] provides a comprehensive analysis of the latest advancements and obstacles encountered in applying machine learning techniques for predicting properties associated with energetic materials. The primary focus is placed on highlighting the considerable potential of machine learning for propelling the development of these materials. In the field of materials science, there has been significant progress in utilizing artificial intelligence (AI) and machine learning (ML) techniques to predict material properties. However, a major difficulty that persists is the lack of comprehensive interpretability in these models, as highlighted by Kondo et al. in their studies [33,34,35]. Although explanation techniques like LIME and SHAP have been employed in several fields, there is a noticeable lack of comprehensive comparative analysis primarily focused on materials science [36,37,38]. Our critical analysis of the current state of the art in materials science reveals several key inconsistencies in the application of machine learning, particularly in ceramic materials. A prominent concern is the lack of transparency and interpretability in these models [39,40], often referred to as “black boxes” due to their reliance on substantial training data [38,39,40]. Despite machine learning’s preference for material property discovery and prediction, there is a crucial need for model outputs to be both highly accurate and interpretable [39,40,41].

### 1.1. Motivation

We believe that the utilization of AI or machine learning approaches in the prediction of the dielectric constant property of PZT (lead zirconate titanate) ceramic materials holds significant importance in materials science applications for many reasons:

- Firstly, the utilization of this technology expedites the process of materials discovery by facilitating the swift screening and optimization of PZT compositions.
- Consequently, this approach substantially diminishes the temporal and financial resources often expended on experimental trials.
- Furthermore, it enables engineers and researchers to customize PZT materials for particular applications, such as sensors and actuators, by precisely adjusting the dielectric characteristics. This, in turn, promotes innovation in several technical domains.
- In general, the utilization of artificial intelligence (AI) for predicting the features of lead zirconate titanate (PZT) has the capacity to bring about a significant transformation in the field of materials science. This is achieved by optimizing research processes, enhancing productivity, and facilitating the creation of customized materials that exhibit enhanced performance characteristics.

### 1.2. Contribution

The primary contribution of this study is the utilization of an interpretable TabNet deep learning approach to forecasting the dielectric constant attribute of PZT materials. This analysis aims to evaluate each parameter’s significance, investigate the connections among these features, and offer justifications for the model’s particular judgments. The study being suggested demonstrates a novel approach within the existing body of literature, presenting a methodology that has yet to be investigated. This signifies the initial execution of the planned concept. As a result, this research endeavor also aims to offer insights into model results by employing diverse SHAP plots.

To summarize, this work presents significant contributions to the field of piezoelectric material property prediction as follows:

- Development of a novel deep learning (DL) framework based on TabNet specifically tailored to predict PZT ceramics’ properties, demonstrating its effectiveness in accurate predictions.
- Investigation into the intricate relationships between the model and predictor variables, particularly under various input parameters, through a comprehensive analysis of individual forecasts using Shapley outputs. This analysis not only enhances model interpretability but also provides insights into the underlying factors affecting ceramic property predictions.
- By developing a specialized deep learning framework based on TabNet, our research enables highly accurate predictions of ceramic properties crucial for advanced materials engineering, providing valuable insights into the predictive relationships and enhancing the precision of model evaluations in the field of piezoelectric material property prediction.
- The proposed study has the potential to provide valuable assistance to materials scientists and engineers in the optimization of production processes for piezoelectric materials. By accurately anticipating important features, this research can contribute to enhanced product performance and efficiency.

## 2. Materials and Methods

**Figure 1:**
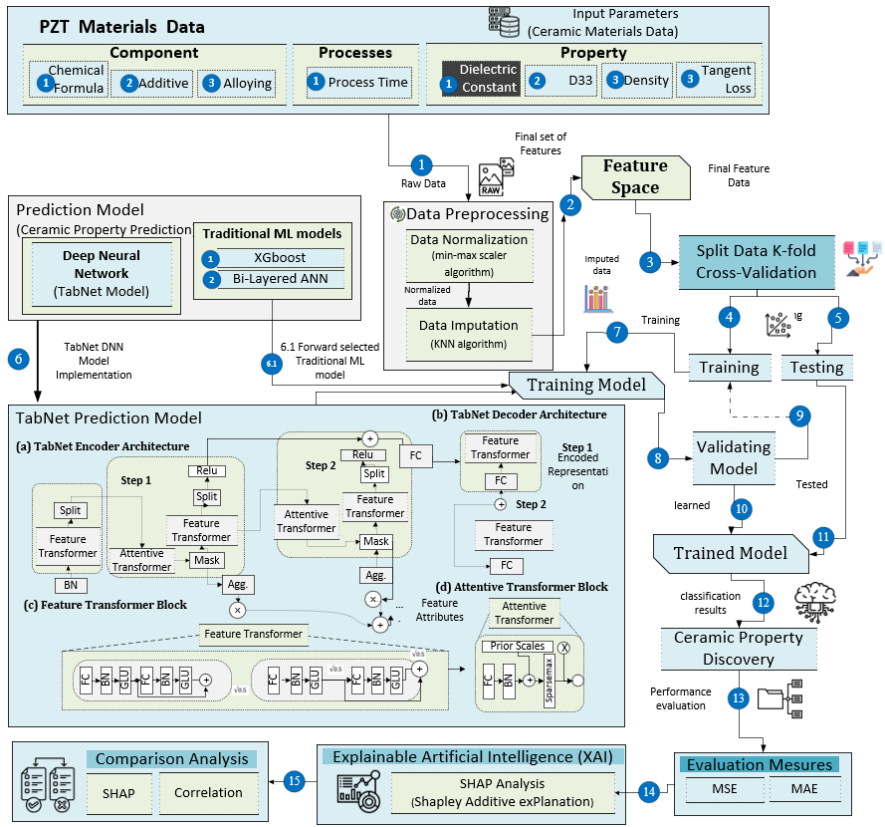
Proposed architecture for explainable AI analysis for identifying influential ceramic parameters using deep learning model.

The field of machine learning has brought about substantial advancements in the areas of classification and prediction across various domains [42,43,44,45], leading to the discovery of novel possibilities and the acquisition of valuable knowledge. This study proposes a comprehensive technique for predicting the dielectric constant property of PZT materials. The detailed architecture of the proposed framework is shown in Figure 1, formally presented in Algorithm 1, and focuses on the following main modules:

- This study utilized the TabNet-based deep learning (DL) [46] approach to construct a model. The inputs for the model included ceramic components and processes, covering host, additive, alloying, and process value. The model underwent training and testing using data related to PZT material processes and components.
- The XAI framework was applied to interpret the results of the TabNet model predictions. This approach facilitated the assessment of the individual impact of each input component on the prediction, ensuring the transparency and comprehensibility of the analysis.
- To bolster the robustness and reliability of the findings, a five-fold cross-validation technique was utilized.
- To assess the effectiveness of the ML model, a performance evaluation was conducted using metrics such as mean squared error (MSE) and mean absolute error (MAE) for visualization of the outcomes.

### 2.1. Dataset

**Figure 2:**
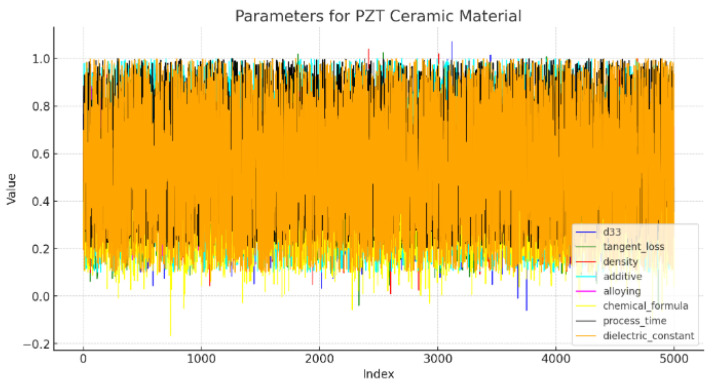
PZT ceramic material parameters, showcasing the diversity and range.

The dataset under consideration consists of around 5000 instances of PZT (lead zirconate titanate) materials and was graciously donated by the Materials Digitalization Center, Korea Institute of Ceramic Engineering and Technology, Republic of Korea, for a collaborative study with Jeju National University. The dataset comprises significant attributes, including material composition, processing parameters, and resultant features. The overview of the dataset values’ trends is shown in Figure 2, wherein additive, alloying, and chemical formulas represent PZT components; process denotes the PZT process and its completion time; d33, tangent loss, density, and dielectric constant denote properties, wherein the symbol “d” represents a piezoelectric charge coefficient. The term “33” denotes a particular mode of the piezoelectric response. Among these parameters, the dielectric constant is the target variable; the rest are input parameters. The detailed explanation of these parameters is illustrated in Table 1.
**Algorithm 1** Shapley-based explainable algorithm for PZT property prediction**Input:** PZT ceramic material components-host, additive, alloying, chemical formula, process time, d33, density, tangent loss; PZT ceramic material dielectric constant.**Output:** Dielectric Constant Property, MSE, and MAE.**Procedure:** 
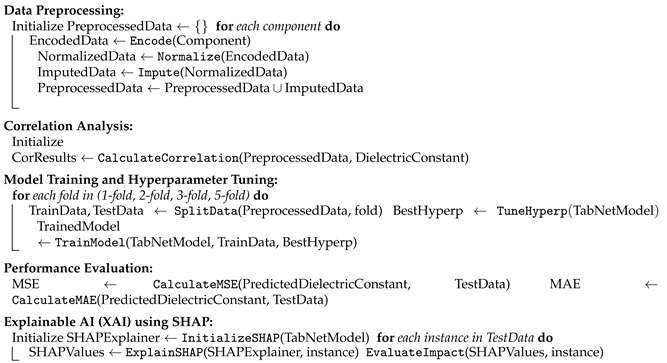
**Comparison Analysis:**  CompareSHAPandCorrelation(SHAPValues, CorrelationResults)

### 2.2. Data Preprocessing

**Figure 3:**
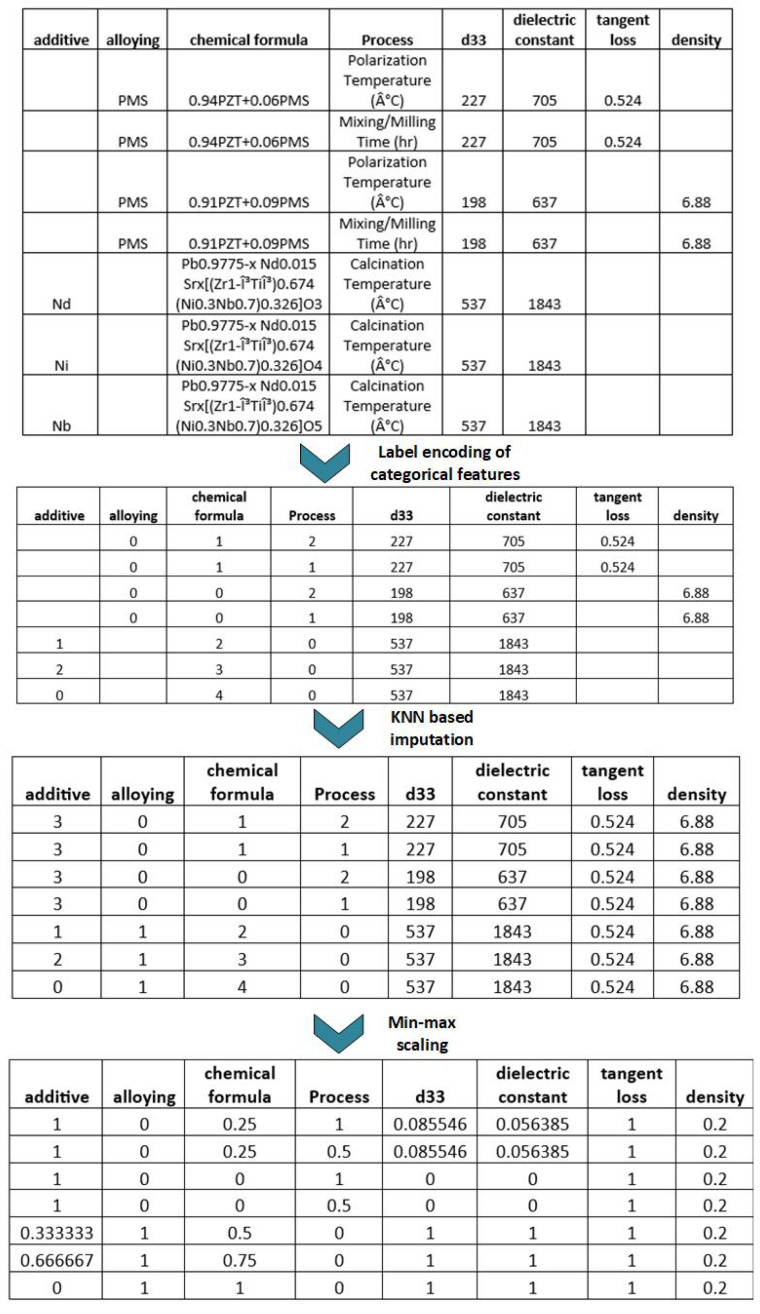
Employed raw PZT material data overview featuring host, additive, alloying, process value, and dielectric constant properties.

In the context of our PZT material property prediction, we utilized data preparation approaches to improve the overall quality of our dataset. Figure 3 presents a thorough data preparation procedure that was implemented on a dataset consisting of PZT (lead zirconate titanate) ceramic materials. The workflow commences with the initial dataset, which encompasses various categories, including additives (such as PMS, Nd, Ni, Nb), alloying information, chemical formulas, and process details. Additionally, the dataset includes attributes such as d33 (representing the piezoelectric coefficient), dielectric constant, tangent loss, and density. The initial step involved the application of label encoding to convert categorical information into numerical values, hence enhancing the computational process. Subsequently, the dataset underwent KNN (K-Nearest Neighbors) [47] imputation techniques to estimate and substitute missing values, perhaps leveraging the characteristics of analogous materials. Afterward, min–max scaling [48] was applied as a means of normalizing numerical data inside a predetermined range, often ranging from 0 to 1. This normalization process has significant importance for numerous machine learning algorithms that exhibit sensitivity towards the scale of the input data. The process of normalization is implemented to provide a uniform scale for all features, hence preventing any individual feature from exerting excessive influence on model training as a result of its scale. The standard formula for normalization is shown in Equation (1).

(1) $$X_{\mathrm{normalized}}=\frac{X_{\max}-X_{\min}}{X-X_{\min}}$$

### 2.3. Detailed Architecture of TabNet Architecture

In this study, we employed a DNN classification model based on TabNet [46] to predict the “dielectric constant” value of PZT materials using the above dataset. The choice of TabNet as the deep learning framework was motivated by its compatibility with tabular data, which aligns well with the nature of our dataset comprising process and component parameters of piezoelectric materials. The selection of TabNet was primarily driven by its performance in the contemporary state of the art [46] and its notable interpretability attributes, including attention processes and Shapley values. The competitive predictive performance and computational efficiency of the model further enhance its eligibility for our research, finally establishing it as the ideal alternative for forecasting ceramic qualities in our study. In addition, TabNet is interpretable, and it can also achieve competitive performance, which is important when you want to make accurate predictions.

Deep neural networks (DNNs) provide a proficient method for encoding tabular data using end-to-end training, diminishing the necessity for substantial feature engineering, particularly when dealing with sizable datasets.

#### 2.3.1. TabNet Decoder Architecture

The TabNet architecture comprises three components: a feature transformer, an attentive transformer, and feature masking implemented at every decision step. This architectural framework is designed to process and analyze category and quantitative data effectively. The output of the attentive transformer in prior iterations has a significant impact on the properties of succeeding iterations, hence playing a crucial role in the whole process of decision making. The complete procedure for predicting the dielectric constant property was implemented by employing Keras and PyTorch, with TensorFlow serving as the foundational framework.

#### 2.3.2. Feature Selection

**Figure 4:**
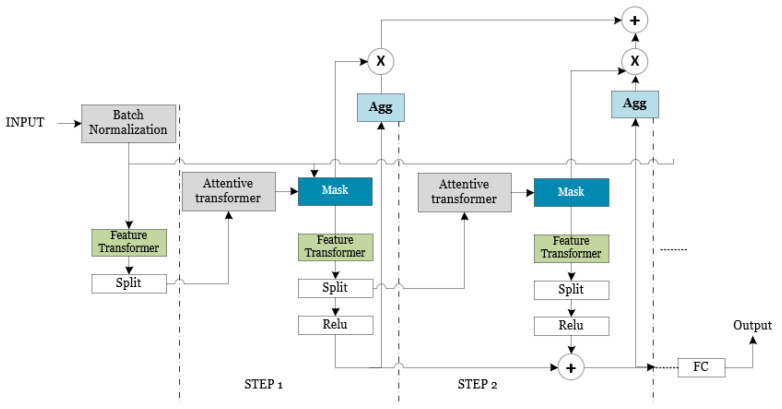
The structural layout of the TabNet encoder module architecture.

**Figure 5:**
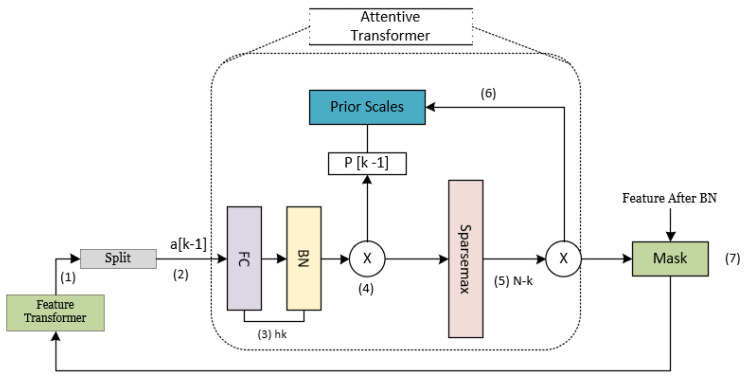
The structural layout of the TabNet attentive transformer layer.

The feature selection process was carried out using the mask module in every decision step, where the attentive converter determines the specific function to be executed. The attentive transformer facilitates the feature selection process in the present decision stage, as illustrated in Figure 4. This is achieved through the acquisition of a mask through learning. The numerical value assigned to each element in Figure 5 represents the sequential arrangement of tensor flow.

#### 2.3.3. Feature Processing

**Figure 6:**
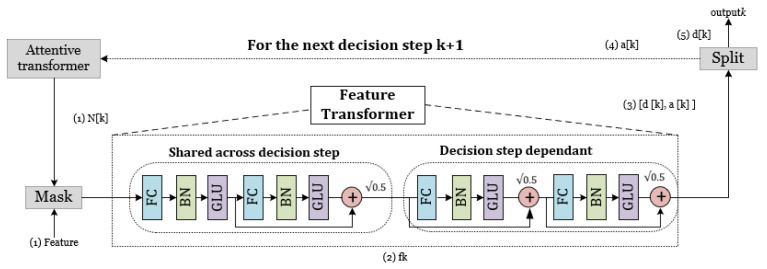
Feature transformer structure module in the TabNet architecture.

The features that have been filtered are thereafter subjected to additional processing through the mask in order to undergo further manipulation within the feature transformer layer. The aforementioned properties are categorized into two distinct components: one component functions as the output for the present phase. In contrast, the other component serves as the input for the subsequent step. The feature transformer layer is composed of three components: the batch normalization (BN) layer, the gated linear unit (GLU) layer, and the fully connected (FC) layer, as depicted in Figure 6.

#### 2.3.4. Decoder Architecture

**Figure 7:**
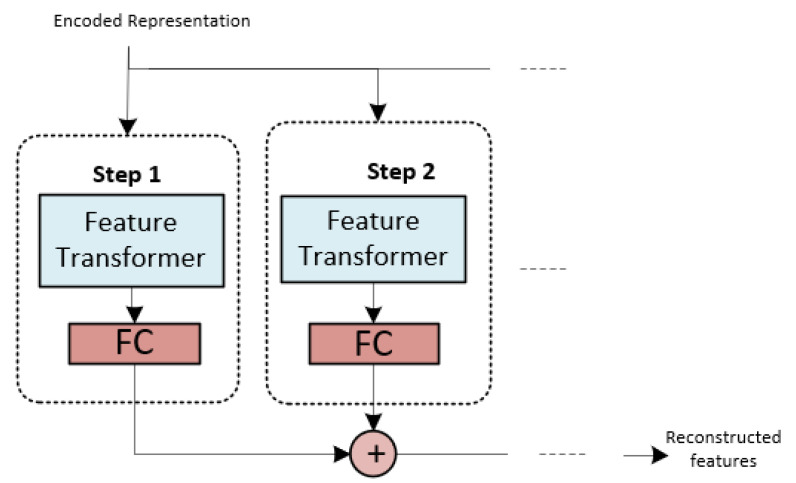
The structural layout of the TabNet decoder module architecture.

The input provided to the decoder comprises the encoded representation depicted in Figure 7, excluding the fully connected (FC) layer. The decoder utilizes the feature transformer layer to reconstruct the representation vector into a feature. A series of processing steps form the reconstructed feature. Each of these core TabNet components is fully implemented to make predictions regarding ceramics’ properties.

### 2.4. Conventional Machine Learning Algorithms

In this study, we utilized Bi-Layered Artificial Neural Network (Bi-Layered ANN) [49] and XGBoost [50] to compare them with the TabNet model. The objective was to evaluate various modeling methodologies and determine the most effective method for forecasting PZT material properties. Through the examination of several algorithmic paradigms, our objective was to determine the optimal and resilient model, hence facilitating thorough assessment and bolstering the dependability of our predictions.

- Bi-layered ANN: Bi-Layered Artificial Neural Network (Bi-Layered ANN) is a specific architecture of an artificial neural network consisting of two distinct layers: an input layer and an output layer. The proposed approach utilizes a network of interconnected nodes, where the connections among nodes are assigned weights. This network is employed to acquire knowledge and establish mappings of intricate relationships in the input data, with the ultimate goal of predicting the material properties of PZT.
- XGboost: In contrast, XGBoost, a type of ensemble learning algorithm, is founded upon decision trees and utilizes a boosting methodology. The proposed methodology involves the iterative and adaptive training process of constructing a series of decision trees. Each subsequent decision tree is designed to rectify the errors made by its predecessor. This sequential approach ultimately leads to the development of a robust predictive model for PZT material properties.

### 2.5. SHAP Interpretable Model

In 2021, Chen [51] proposed Shapley additive explanations (SHAP), an approach rooted in game theory that aims to assess the effectiveness of prediction systems. In order to establish a method that is easily understandable, SHAP utilizes an additive feature attribution strategy, which involves expressing the model’s output as a linear mixture of input variables. The solid theoretical foundations of SHAP make this approach particularly helpful in supervised situations:

- The alignment between the explanation technique and the primary model’s findings is crucial for achieving local accuracy.
- The explanation method should effectively address the issue of missing features by discarding any characteristics that are not present in the primary input.
- The maintenance of consistency is of utmost importance in order to ensure that the significance of a variable remains constant, even when the model’s reliance on said variable is modified, irrespective of the relevance of other variables.

### 2.6. Data Split

**Figure 8:**
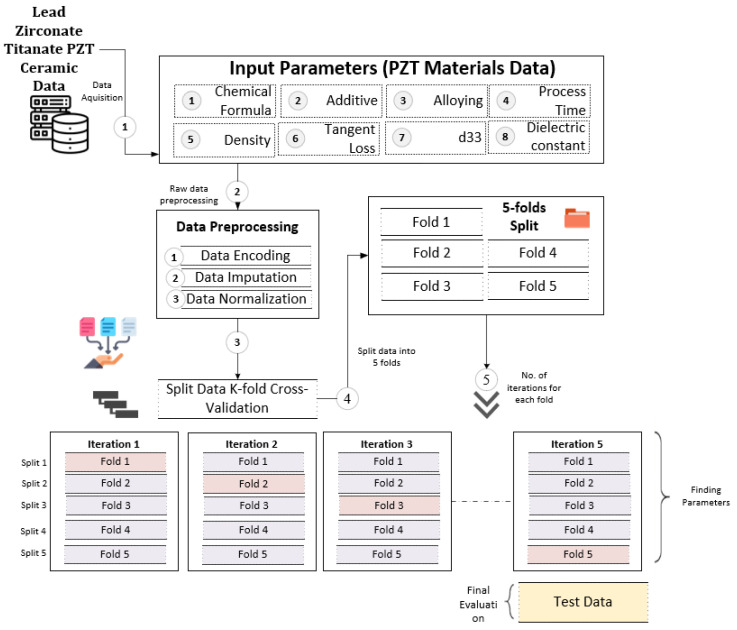
Five-fold cross-validation (CV) technique to improve model prediction performance.

Conventional machine learning techniques follow a systematic process involving model creation with a designated training dataset and later using this model for predictions, such as ceramic property prediction. However, using inadequate training and test datasets can yield unreliable and scientifically inconclusive ML results. To improve the reliability of model performance evaluation, it is recommended to employ a hold-out dataset along with cross-validation (CV) techniques. CV helps mitigate dataset biases and prevents overfitting or underfitting in ML algorithms during the optimization phase. We applied this procedure to preprocess the dataset for training and evaluating our proposed deep learning (DL) models. Our proposed approach underwent five iterations, with one fold being reserved for validation in each iteration, remaining unchanged during training. As a result, the model was trained with 80% of the PZT material data during each iteration, with the performance evaluation being conducted on the remaining 20% of the data. Figure 8 illustrates the utilization of the five-fold CV technique on the employed PZT material inventory dataset.

### 2.7. Evaluation

To assess the performance of the prediction outcomes, we employed standard evaluation measures, including MAE and MSE, which are explained below.

#### 2.7.1. Mean Absolute Error

Mean absolute error, an alternative to MSE, captures the average absolute differences between predicted and actual values. Significantly less sensitive to outliers than MSE, MAE offers a more balanced evaluation, assigning equal weight to errors of all magnitudes. Lower MAE values signify better model performance, making it a suitable metric when seeking robustness against extreme values. The MAE computation formula is shown in Equation (2).

(2) $$MAE=\frac{1}{n}\sum_{i=1}^{n}\left|y_{i}-{\hat{y}}_{i}\right|$$

#### 2.7.2. Mean Squared Error

This is the average of the squared differences between predicted and actual values. Its sensitivity to larger errors is significant, attributing greater weight to them. Lower MSE values suggest superior model performance, yet it is essential to recognize its vulnerability to outliers, where substantial errors can disproportionately influence the overall score. The formula to compute MSE is shown in Equation (3).

(3) $$MSE=\frac{1}{n}\sum_{i=1}^{n}{\left(y_{i}-{\hat{y}}_{i}\right)}^{2}$$

## 3. Performance Analysis

This section presents a comprehensive investigation of the predictive modeling of the dielectric constant property of PZT materials, offering a detailed and nuanced examination. The analysis commences by scrutinizing the concept of “Data Distribution”, offering valuable insights into the fundamental properties of the dataset. In the following, the objective of “Correlation Analysis” is to reveal the complex interactions that exist among the variables. The study titled “Comparative Impact Assessment of Imputation on Analysis Outcomes” examines the consequences of employing data imputation methods on the predictive outcomes. Following that, a comprehensive analysis titled “Comparative Evaluation of TabNet and Conventional Machine Learning Models” outlines the advantages and disadvantages of advanced deep learning in comparison to traditional methodologies. The publication titled “SHAP Analysis Outcomes” provides a comprehensive analysis of the model predictions, offering valuable insights into the interpretation of feature contributions, thus enhancing our comprehension of the model’s performance. Table 2 provides a comprehensive overview of the development environment and hardware specs employed in our research. The implemented methodology utilized TabNet version 3.1, which was implemented using Python 3.8 programming language. The essential libraries utilized in this study encompassed TensorFlow 2.4, scikit-learn 0.24, pandas 1.2, and NumPy 1.19, thereby establishing a resilient and effective computational framework. The system configuration included of an Intel Core i7-10700K central processing unit (CPU) and an NVIDIA GeForce RTX 3080 graphics processing unit (GPU), complemented by 32 gigabytes of DDR4 random-access memory (RAM). The integration of software and hardware played a crucial role in effectively managing the computing requirements of the TabNet method and guaranteeing the replicability of our research.

### 3.1. Data Distribution

**Figure 9:**
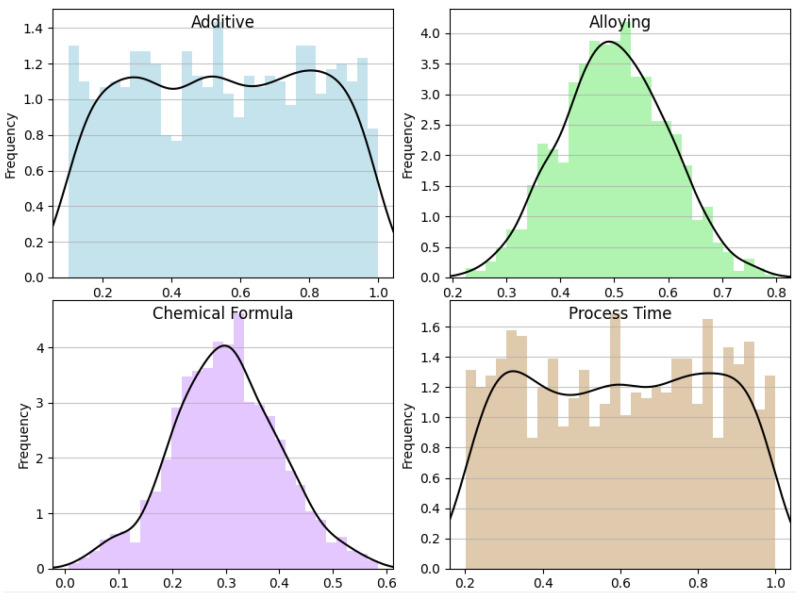
Additive, alloying, chemical formula, and process time data distribution analysis.

**Figure 10:**
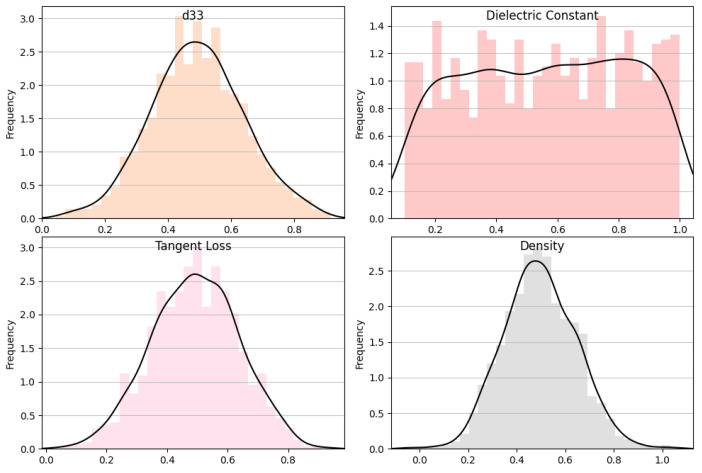
d33, dielectric constant, tangent loss, and density data distribution analysis.

The histograms and overlaid density plots for several variables related to material properties are shown in Figure 9 and Figure 10. “Dielectric constant” is shown as an outcome variable in a wide range of values, while “additive”, “alloying”, “chemical formula”, “process time”, “d33”, “tangent loss”, and “density” are input parameters, each with its own distribution. Most input parameters exhibit a clear central tendency, implying that they are likely controlled during the experimental process, whereas “dielectric constant” shows greater variance, suggesting it is a result of the interplay of different input parameters. The density plots suggest that while some parameters, like “alloying” and “density”, follow a normal distribution, others, like “process time”, might have a more complex distribution, indicative of different underlying processes or a combination of effects.

### 3.2. Correlation Analysis

**Figure 11:**
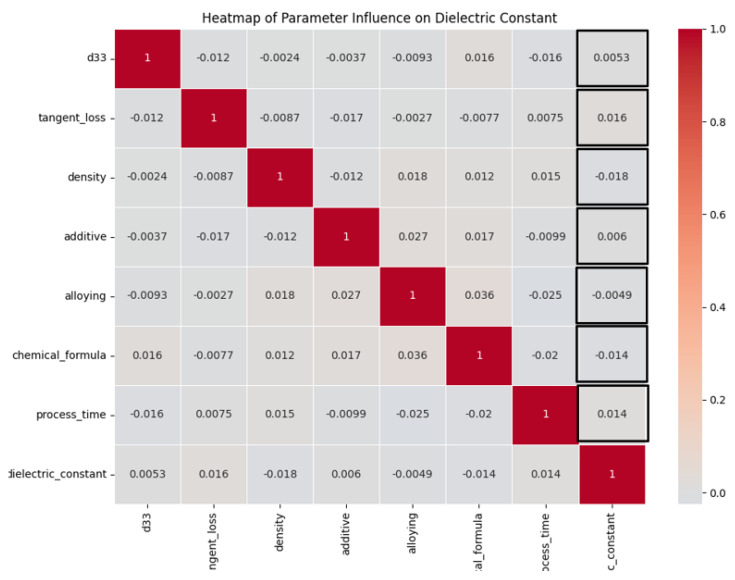
Pearson correlation analysis between input and target parameter.

A heatmap is utilized to visually represent the correlations between many input factors and the dielectric constant, which serves as the dependent variable, as shown in Figure 11. Every individual cell within the dataset denotes the correlation coefficient, which quantifies the strength and direction of the relationship between two variables. The correlation coefficient is a numerical measure that ranges from −1 to 1, indicating the extent to which the variables are linearly related. Values that are close to 1 or −1 imply a robust positive or negative linear relationship, respectively, whilst values in proximity to 0 reflect the absence of a linear association. Within this particular context, it is observed that the variable “d33” has a noteworthy positive correlation with the dielectric constant, suggesting that it holds substantial predictive value. Additional input factors, namely, “tangent loss”, “density”, “additive”, “alloying”, and “chemical formula”, exhibit varying degrees of association, although none are as significant as the correlation observed with “d33”. The variable “process time” demonstrates a modest negative correlation, suggesting a nuanced or potentially indirect association with the dielectric constant. The presented heatmap offers a detailed depiction of the potential impact of each input parameter on the target variable. It is observed that the parameter “d33” exhibits the highest level of influence within the dataset.

### 3.3. Comparative Impact Assessment of Imputation on Analysis Outcomes

This section provides an overview of the anticipated results pertaining to the dielectric constant property. In the first step, we analyze the outcomes derived from a five-fold cross-validation procedure, taking into account two scenarios: one using imputed data and the other without imputed data. Following this, we present a comparison analysis that juxtaposes the performance of the TabNet architecture with that of typical machine learning models over all validation folds.

A comparison analysis is undertaken in order to evaluate the influence of data imputation on the performance of the model. The number of missing records in all the parameters in the PZT material dataset is shown in Table 3. Following that, we conducted two sets of experiments: one utilizing the incomplete dataset, in which occurrences containing missing values were omitted, and another utilizing the dataset after K-Nearest Neighbors (KNN) imputation. By employing this methodology, we were able to quantitatively assess the impact of imputation by conducting a comparative analysis of model performance indicators, including loss, mean absolute error (MAE), and mean squared error (MSE), across the two datasets. The forthcoming discussion will provide a comprehensive analysis of the results, shedding light on the impact of KNN-imputed data on the predictive accuracy of the model. This will offer valuable insights into the effectiveness and reliability of our data-handling approach.

**Figure 12:**
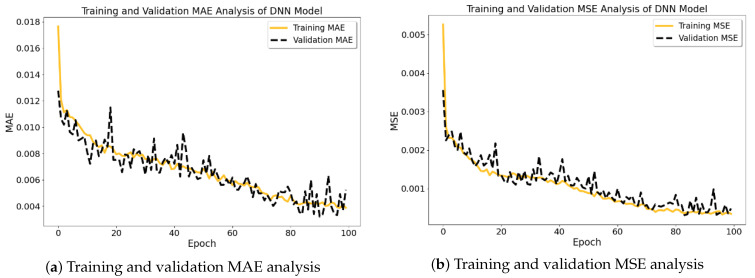
TabNet model prediction outcomes with removed missing records.

**Figure 13:**
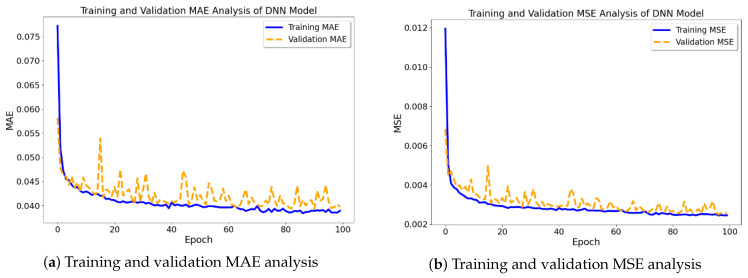
TabNet model prediction outcomes with imputed missing records.

The prediction performance analysis of the TabNet model on two distinct datasets is shown in Figure 12 and Figure 13. One dataset was processed by removing missing records, while the second dataset underwent missing-data imputation using the K-Nearest Neighbors (KNN) method.

In the initial scenario (see Figure 12), whereby the exclusion of missing records was implemented, the model’s training and validation MAE (mean absolute error) and MSE (mean squared error) demonstrate a degree of consistent performance after an initial phase. Nevertheless, in the second scenario (see Figure 13), employing KNN imputation yields a discernible enhancement in the rate of early convergence, indicating that the imputation technique potentially plays a role in fostering a more steadfast and precise learning process. The graphs illustrate that the utilization of KNN imputation contributes to a decrease in the disparity between training and validation metrics. This effect is particularly evident in the mean squared error (MSE) graph, where the lines representing training and validation metrics exhibit greater proximity in comparison to the dataset including removed instances. This implies that the model that was trained using the imputed dataset has the potential to exhibit improved generalization and hence provide more dependable predictions.

**Figure 14:**
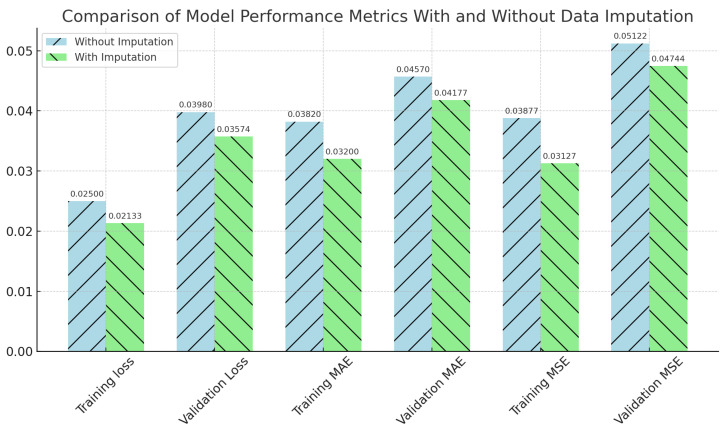
Comparison of model performance metrics with and without data imputation.

Similarly, Figure 14 and Table 4 present the values pertaining to model performance measures, considering both the scenarios of data imputation and non-imputation. The bars are shaded in a light-blue hue to represent the scenario without imputation, while a light-green hue is used to depict the scenario with imputation. Slashes and backslashes are utilized as patterns to differentiate between the two scenarios. The visual comparison presented herein provides obvious evidence that the model trained with imputed data exhibits superior performance. This is evident from the lower values seen for training loss, validation loss, mean absolute error (MAE), and mean squared error (MSE). These findings suggest that the process of data imputation has a beneficial influence on the correctness of the model.

### 3.4. Comparative Evaluation of TabNet and Conventional Machine Learning Models

**Figure 15:**
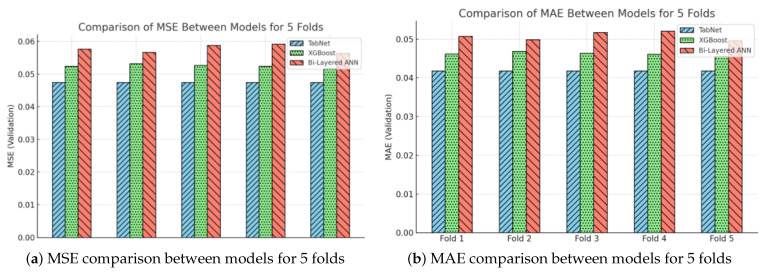
Comparative evaluation of TabNet and conventional machine learning models.

The bar charts shown in Figure 15a,b depict a comparative assessment of the mean squared error (MSE) and mean absolute error (MAE) for three distinct predictive models across five validation rounds. TabNet consistently demonstrates superior performance compared with the other models, as evidenced by its consistently lower mean squared error (MSE) and mean absolute error (MAE) values. This suggests a greater level of prediction accuracy and model reliability. The XGBoost model demonstrates superior performance in terms of error metrics compared with TabNet. However, it still outperforms the Bi-Layered ANN model, which exhibits the greatest error rates throughout the folds. The observed hierarchy of model performance indicates the efficacy of TabNet in effectively managing the given dataset and its inherent intricacies. The visual distinction of each fold inside the bars allows for the clear differentiation of the variability in and consistency of the performance exhibited by each model across various subsets of data.

### 3.5. Shapely Analysis of Dielectric Constant Property Prediction Model

**Figure 16:**
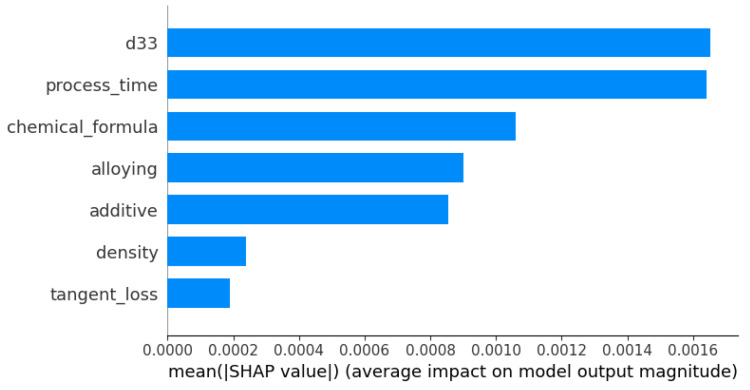
Mean SHAP values illustrating the average impact of model features on output magnitude.

This section provides an in-depth analysis of the SHAP (Shapley additive explanations) outcomes derived from the TabNet model, focusing on the prediction of the dielectric constant property of PZT materials. The aim is to meticulously evaluate the contribution and significance of each parameter within the model’s predictive framework. The bar chart shown in Figure 16 shows the mean SHAP values assigned to different features within a predictive model. These values serve to quantify the average influence of each feature on the model’s output. The amount of average impact is shown by the length of the bars, where larger bars signify a higher level of influence. Within the depicted picture, the feature denoted by “d33” exhibits the greatest mean SHAP value, indicating its predominant positive influence on the model predictions. Additionally, the features “process time”, “chemical formula”, “alloying”, “additive”, “density”, and “tangent loss” are presented in descending order based on their beneficial impact.

**Figure 17:**
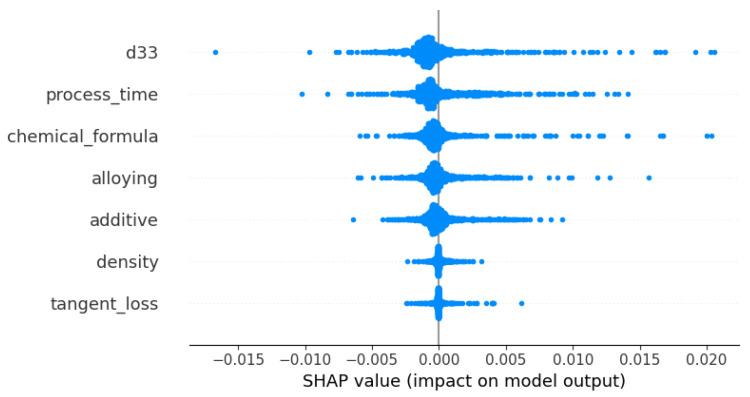
Beeswarm plot of SHAP values for each feature, highlighting the distribution and density of impact on the model’s output.

The beeswarm plot shown in Figure 17 effectively portrays the distribution of SHAP values pertaining to each feature across the entirety of data points within the model. Each data point is represented by a dot, where the intensity of color and the dispersion of the dots indicate the frequency and range of influence of the respective feature. The feature denoted by “d33” exhibits a broad dispersion of positive SHAP values, indicating a substantial and fluctuating impact on the output of the model. The feature “process time” also demonstrates a range of values, albeit with a lower density in comparison to “d33”, suggesting a smaller yet still significant impact on the result.

**Figure 18:**
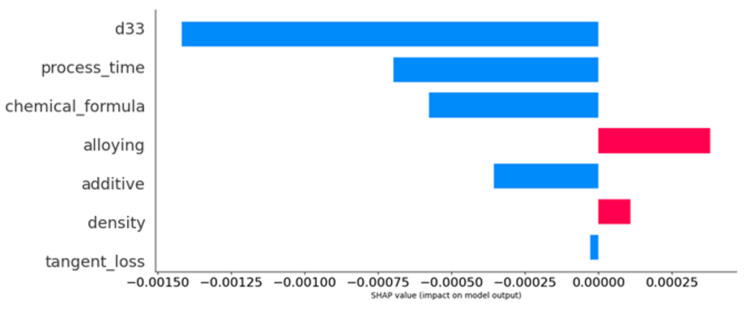
Bar chart of average SHAP values of model features, with positive impacts in blue and negative ones in pink, demonstrating the relative influence on model output.

Similarly, the bar chart shown in Figure 18 indicates the average impact of each feature on the model output, measured in SHAP values. Positive contributions are shown in blue, and negative contributions, in pink. Notably, “d33” shows the largest positive impact, whereas “tangent loss” has a marginal negative impact. The varying bar lengths represent the relative significance of each feature, with “process time” and “chemical formula” demonstrating moderate positive impacts, and “alloying”, “additive”, and “density” showing smaller yet positive contributions.

**Figure 19:**
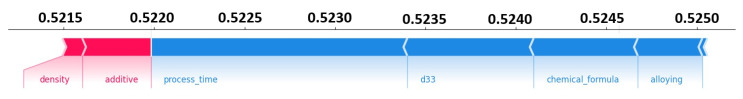
Force plot depicting individual feature contributions.

The force plot shown in Figure 19 shows the distinct influences exerted by each feature on a particular model prediction. The plot demonstrates the impact of each feature’s value on the model’s output, as it transitions from the base value (representing the average model output across the dataset, depicted on the left side of the plot) to the final prediction (displayed on the right side). The figure displays features that positively influence the forecast in blue, while features that negatively impact the prediction are represented in pink. The primary factors positively influencing the forecast value are “d33”, “chemical formula”, and “alloying”, whilst “density” and “additive” have a minor negative impact.

**Figure 20:**
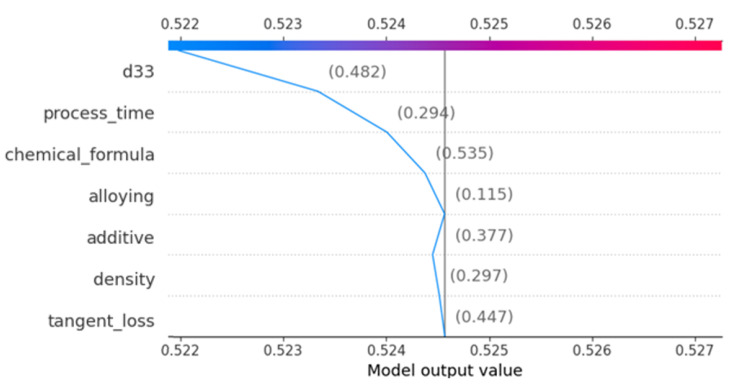
Waterfall plot demonstrating the cumulative impact of features on prediction.

The waterfall plot shown in Figure 20 presents the incremental impact of individual features on a certain model prediction. The narrative commences by establishing an initial projected model output value, which represents the average forecast throughout the dataset. Subsequently, the influence of each feature is incrementally incorporated or deducted based on the respective SHAP value. The variable “d33” has a notable positive impact, whereas the variable “process time” has a negative effect. The numbers enclosed in parenthesis depict the intermediate forecasts following the contribution of each feature, up to the ultimate anticipated value emphasized at the conclusion of the plot.

**Figure 21:**
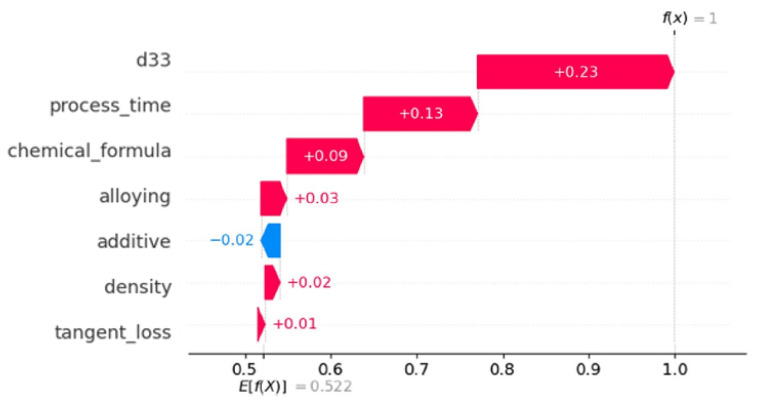
Waterfall plot of feature contributions to a model prediction.

The waterfall plot shown in Figure 21 delineates the individual contributions of various features towards a certain prediction generated by the model. The visualization demonstrates the progressive impact of individual features, commencing from the anticipated value (E[f(X)]) and then adjusting the prediction (f(X) = 1) by incorporating the corresponding SHAP value of each feature. The analysis reveals that the variables “d33” and “process time” exhibit significant positive effects on the prediction, whilst the variable “additive” has a minor negative impact.

**Figure 22:**
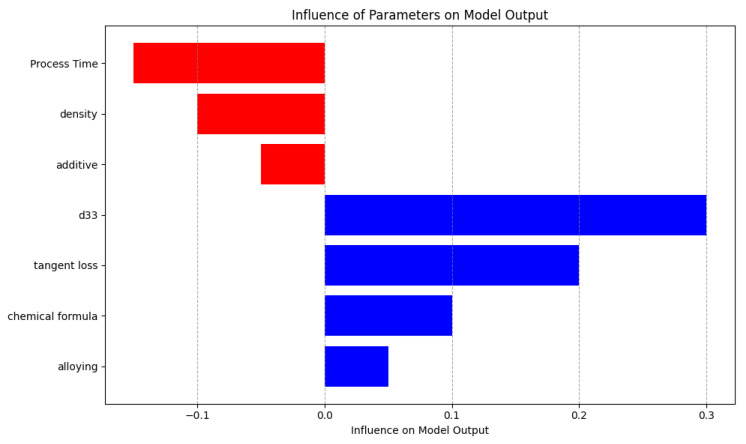
Comparative impact of parameters on model output.

The bar chart shown in Figure 22 shows the varying effects of different parameters on the output of a model. Red bars extending to the left symbolize negative influences, with the variable “process time” exhibiting the most significant adverse impact. On the contrary, the presence of positive influences is shown by blue bars that expand towards the right side of the graph. Among these influences, it is noteworthy that “d33” has the most significant positive effect. The visual contrast serves to emphasize the elements that have the highest predictive capacity in either augmenting or diminishing the output values of the model.

### 3.6. Factors Affecting the Dielectric Constant

- *D33*: The d33 coefficient emerges as the most influential parameter impacting the dielectric constant, as seen by the highest positive SHAP values. The metric that quantifies piezoelectric strain is strongly correlated with the dielectric behavior, highlighting its substantial advantageous influence.
- *Process time*: In contrast to the findings of d33, it was discovered that process time exerts a significant negative influence on the dielectric constant. Extended processing durations have a notable influence, albeit adverse in this particular case, on the dielectric characteristics, potentially leading to improved crystallinity or phase purity.
- *Chemical formula*: The SHAP study demonstrates that the chemical formula has a significant positive influence. The inherent qualities of a substance, such as permittivity, are determined by the precise composition and quantity of its components, thus highlighting the vital role they play.
- *Alloying*: The process of alloying demonstrates a discernible beneficial influence on the piezoelectric properties, hence changing the dielectric constant in a significant manner. The incorporation of supplementary elements has been recognized as a substantial catalyst in improving the performance of a material.
- *Additive*: Additives, although they possess a certain degree of influence, exhibit comparatively reduced magnitude of impact when juxtaposed with the underlying composition of the material. The SHAP values indicate a slight negative impact, indicating that additives may be involved in modifying microstructural properties such as grain size.
- *Density*: The relationship between density and the dielectric constant is less straightforward compared with its influence on mechanical qualities.

### 3.7. Comparison Analysis between Correlation and Shapely

After performing an examination of the results obtained from both SHAP values and correlation analysis, it becomes apparent that there exist disparities in the significance of parameters between these two approaches. The SHAP analysis reveals that the variables d33, tangent loss, and chemical formula exhibit a noteworthy positive impact on the output of the model. Conversely, the variable process time demonstrates a detrimental effect on performance. On the other hand, the correlation analysis indicates complex and interrelated associations among the parameters, wherein the same components do not consistently demonstrate significant individual correlations with the target variable. This suggests that although certain parameters are considered significant in both analyses, there are variations in the extent and type of their significance. SHAP primarily emphasizes the predictive influence of parameters, whereas correlation analysis primarily examines the correlations among variables.

## 4. Practical Implementation and Use Cases

The proposed study can be implemented in various real-world applications as explained below:

Design and production:

- The incorporation of Computer-Aided Design (CAD) systems enables the enhancement of material selection for the purpose of optimizing the design of piezoelectric devices.
- Real-time quality control is a crucial aspect of manufacturing lines, as it involves the continuous adjustment of process parameters to guarantee that the material qualities align with the specified design specifications.

Materials development:

- This study aims to provide guidance for experimental design in the research and development (R&D) of novel piezoelectric materials by utilizing predictive models to estimate attributes based on compositional data.

Supply chain management:

- The practice of strategically stocking materials in supply chains is informed by the use of predictive analytics to forecast market demands and assess product performance requirements.

Sustainability:

- The objective is to identify materials that effectively balance performance attributes while also minimizing their environmental impact, thus aligning with the principles of eco-friendly design.

Education and compliance:

- This study aims to provide training tools specifically designed for engineers and materials scientists, with the purpose of facilitating the application of artificial intelligence (AI) in the prediction and selection of material properties.

## 5. Conclusions

This paper conducted a thorough examination of the prediction capacities of different models, including TabNet, Bi-Layered ANN, and XGBoost, in relation to the crucial objective of predicting the dielectric constant property of PZT ceramics. The findings of our study, which involved meticulous experimentation and thorough performance analysis, are here presented. Significantly, TabNet demonstrated superior performance compared with both Bi-Layered Artificial Neural Network (ANN) and XGBoost, hence highlighting its effectiveness in capturing complex patterns present in the dataset. The incorporation of Shapley additive explanations (SHAP) into our research yielded significant insights into the influential aspects that contribute to the prediction of the dielectric constant. The SHAP plots demonstrate that both process values and additives exerted considerable impact, hence highlighting their importance in achieving precise property predictions. On the other hand, the host material exhibited relatively diminished influence on the resulting dielectric constant. The comprehensive understanding obtained through the utilization of SHAP analysis not only improved the interpretability of our models but also provided valuable insights for materials scientists and researchers seeking to optimize PZT ceramics for particular applications. The results of our research, which culminated in the exceptional performance of TabNet and the detailed insights obtained from SHAP analysis, have significantly advanced our understanding and predictive abilities in the field of PZT ceramics. The incorporation of sophisticated machine learning models and interpretable analysis tools constitutes a notable advancement in the pursuit of accurate and transparent predictions in the field of materials science. The observed results show potential for further progress in the field of materials discovery and emphasize the crucial significance of explainable artificial intelligence in revealing the complexities of material properties.

## Figures and Tables

**Table 1:** Explanation of PZT material features.

Sr #	Feature Name	Category	Description
1	Additive	Component	Additives incorporated into PZT (lead zirconate titanate) materials serve as supplementary components that are introduced to alter or augment the material’s properties, hence rendering it acceptable for particular applications.
2	Alloying	Component	The process of alloying in PZT materials involves incorporating additional elements or metals to modify and improve the material’s characteristics, such as electrical conductivity or mechanical strength, to cater to certain applications.
3	Chemical formula	Component	The chemical formula utilized in PZT materials denotes the precise amalgamation of components and their respective proportions, commonly denoted as Pb(Zr_x_Ti_1−x_)O_3_. The variable x inside the formula specifies the ratio of Zr to Ti, influencing the material’s qualities.
4	Process value	Process	Within the context of PZT materials, the term “process” pertains to the stages involved in their manufacturing, encompassing activities such as mixing and sintering. Conversely, “process time” denotes the time required to complete these stages. The duration of the processing period has a significant influence on the quality and characteristics of the material. The process value represents the amalgamation of both factors.
5	D33	Property	The feature denoted by “d33” refers to a piezoelectric characteristic that quantifies the response of a material to mechanical stress exerted in a direction perpendicular to its electric field. The term “d33” denotes the 3-3 mode, which specifically refers to measuring a material’s piezoelectric response when subjected to stress perpendicular to its electric field.
6	Dielectric constant	Property	The dielectric constant, commonly represented as “ε” or “k”, quantifies the ability of a substance to store electrical energy within an electric field. PZT materials are important in various applications, such as capacitors, sensors, and transducers.
7	Tangent loss	Property	The tangent loss, also known as “tan δ” is a measure of the amount of energy dissipated in a dielectric material during the oscillation of an electric field. Using PZT materials with low tangent loss is highly advantageous in various applications such as sensors and actuators, since it enables effective energy utilization.
8	Density	Property	The concept of density in PZT materials refers to measuring mass per unit volume. Mechanical strength and performance are crucial factors in certain applications, such as sensors and actuators, where a preference is generally given to increased density.

**Table 2:** Development environment and hardware specifications.

Category	Description
Method and version	TabNet 3.1 in Python
Programming environment	Python 3.8, key libraries: TensorFlow 2.4, Keras 2.4, PyTorch 1.7, scikit-learn 0.24, pandas 1.2, NumPy 1.19
Hardware specifications	CPU: Intel Core i7-10700K, GPU: NVIDIA GeForce RTX 3080, RAM: 32 GB DDR4

**Table 3:** Number of missing records for each parameter.

Sr #	Parameter	Missing Records	Sr #	Parameter	Missing Records
1	Additive	205	5	Density	200
2	Alloying	79	6	d33	35
3	Chemical formula	321	7	Tangent loss	24
4	Process Time	185	8	Dielectric constant	101

**Table 4:** Comparison of model performance metrics with and without imputation.

Metric	Without Imputation	With Imputation
Training MAE	0.03820	0.03200
Validation MAE	0.04570	0.04177
Training MSE	0.03877	0.03127
Validation MSE	0.05122	0.04744

## Data Availability

The data featured in this study can be obtained upon request from the corresponding author. The data is not publicly accessible due to its confidential nature, as it is an integral part of an ongoing project.
